# Supplementary material for: Content, Mechanism, and Outcome of Effective Telehealth Solutions for Management of Chronic Obstructive Pulmonary Diseases: A Narrative Review
Source: Healthcare (Basel). 2023 Dec 14;11(24):3164. doi: 10.3390/healthcare11243164 (PMC10742533; doi:10.3390/healthcare11243164)

Supplementary file 1. Search strategy example from PubMed up to October 2023.

Search number	Query	Search Details	Results
1	telehealth	"telehealth s"[All Fields] OR "telemedicine"[MeSH Terms] OR "telemedicine"[All Fields] OR "telehealth"[All Fields]	64,533
2	digital health	"mayo clin proc digit health"[Journal] OR "lancet digit health"[Journal] OR "eur heart j digit health"[Journal] OR "digit health"[Journal] OR ("digital"[All Fields] AND "health"[All Fields]) OR "digital health"[All Fields]	66,692
3	#1 or #2	"telehealth s"[All Fields] OR "telemedicine"[MeSH Terms] OR "telemedicine"[All Fields] OR "telehealth"[All Fields] OR ("mayo clin proc digit health"[Journal] OR "lancet digit health"[Journal] OR "eur heart j digit health"[Journal] OR "digit health"[Journal] OR ("digital"[All Fields] AND "health"[All Fields]) OR "digital health"[All Fields])	124,937
4	COPD	"pulmonary disease, chronic obstructive"[MeSH Terms] OR ("pulmonary"[All Fields] AND "disease"[All Fields] AND "chronic"[All Fields] AND "obstructive"[All Fields]) OR "chronic obstructive pulmonary disease"[All Fields] OR "copd"[All Fields]	108,260
5	#3 and #4	("telehealth s"[All Fields] OR "telemedicine"[MeSH Terms] OR "telemedicine"[All Fields] OR "telehealth"[All Fields] OR ("mayo clin proc digit health"[Journal] OR "lancet digit health"[Journal] OR "eur heart j digit health"[Journal] OR "digit health"[Journal] OR ("digital"[All Fields] AND "health"[All Fields]) OR "digital health"[All Fields])) AND ("pulmonary disease, chronic obstructive"[MeSH Terms] OR ("pulmonary"[All Fields] AND "disease"[All Fields] AND "chronic"[All Fields] AND "obstructive"[All Fields]) OR "chronic obstructive pulmonary disease"[All Fields] OR "copd"[All Fields])	1,190
6	#3 and #4	(("telehealth s"[All Fields] OR "telemedicine"[MeSH Terms] OR "telemedicine"[All Fields] OR "telehealth"[All Fields] OR ("mayo clin proc digit	184

		health"[Journal] OR "lancet digit health"[Journal] OR "eur heart j digit health"[Journal] OR "digit health"[Journal] OR ("digital"[All Fields] AND "health"[All Fields]) OR "digital health"[All Fields])) AND ("pulmonary disease, chronic obstructive"[MeSH Terms] OR ("pulmonary"[All Fields] AND "disease"[All Fields] AND "chronic"[All Fields] AND "obstructive"[All Fields]) OR "chronic obstructive pulmonary disease"[All Fields] OR "copd"[All Fields])) AND (clinicaltrial[Filter])	

Supplementary file 2. Number of published clinical trials per year up to October 2023.

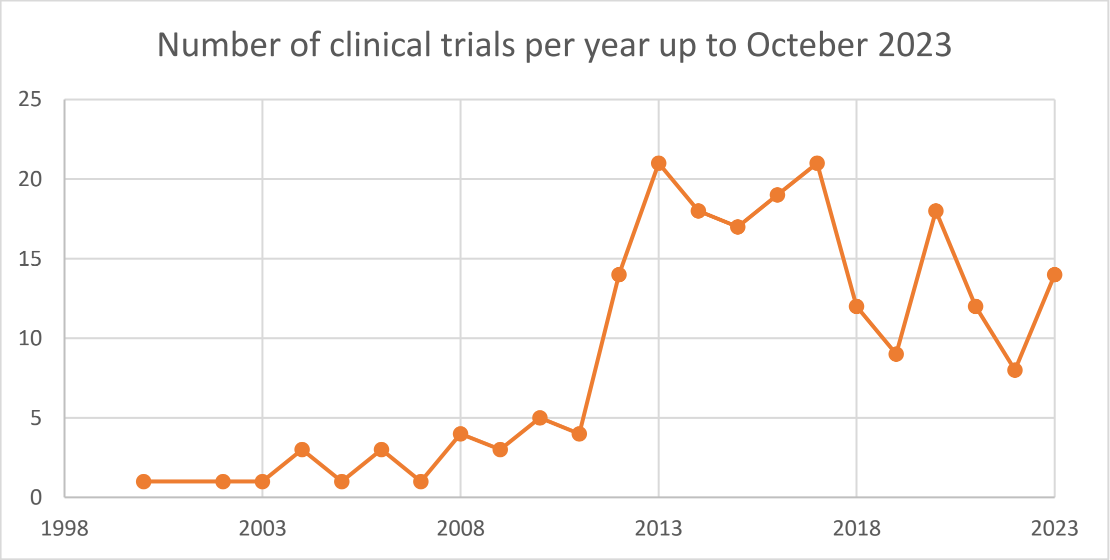

Supplement: Supplementary file 1 [file healthcare-11-03164-s001.zip › healthcare-2696924-supplementary.pdf]
